# Biopsy-proven IgG4-related lung disease

**DOI:** 10.1186/s12890-016-0181-9

**Published:** 2016-01-25

**Authors:** Xuefeng Sun, Hongrui Liu, Ruie Feng, Min Peng, Xiaomeng Hou, Ping Wang, Hanping Wang, Wenbing Xu, Juhong Shi

**Affiliations:** Department of Respiratory Medicine, Peking Union Medical College Hospital, 100730 Beijing, China; Department of Pathology, Peking Union Medical College Hospital, Beijing, China

**Keywords:** Immunoglobulin G4, Lung, Pathology, Computed tomography

## Abstract

**Background:**

Immunoglobulin G4-related disease (IgG4-RD) is a fibroinflammatory disorder that may involve single or multiple organs. Biopsy-proven lung involvement of this disease is occasionally reported, but not well understood.

**Methods:**

Patients with the diagnosis of biopsy-proven IgG4-related lung disease (IgG4-RLD) from Peking Union Medical College Hospital between January 2011 and July 2015 were retrospectively analyzed. Age, sex, clinical symptoms, laboratory findings, pulmonary function test results, chest CT tests, positron emission tomography (PET) examinations, treatments and prognoses were retrieved from medical records and analyzed.

**Results:**

Seventeen patients were included in this study (mean age: 44.8 ± 15.0 years). Ten patients were diagnosed via surgery, and 7 patients were diagnosed via percutaneous transthoracic core-needle lung biopsy. Extrapulmonary involvement was observed in only one patient. The clinical symptoms included cough, fever, dyspnea, chest pain and hemoptysis. The serum IgG4 concentration was elevated in 7/13 patients (mean: 1955 ± 1968 mg/L). The chest CT findings included mainly nodules and masses with spiculated borders, alveolar consolidations with air bronchograms, and ground glass opacities with or without reticular opacities. PET scans indicated increased standardized uptake values, and 7/8 patients were correctly diagnosed with benign inflammation. Corticosteroids and immunosuppressants were administered to 14/17 patients and effectively alleviated the disease.

**Conclusions:**

In biopsy-proven IgG4-RLD, a normal serum IgG4 concentration is commonly seen, while extrapulmonary involvement is infrequent. Alveolar consolidation with air bronchograms is an important imaging finding of IgG4-RLD, which has not been emphasized before.

## Background

Immunoglobulin G4 (IgG4)-related disease (IgG4-RD) is a recently recognized fibroinflammatory condition that is characterized by tumefactive involvement of single or multiple organs, marked lymphoplasmacytic infiltrates and fibrosis with abundant IgG4-positive plasma cells. IgG4-RD is often associated with elevated serum IgG4 concentrations [1]. Since Hamano and colleagues (2001) first reported elevated serum IgG4 concentration in autoimmune pancreatitis [2], IgG4-RD has been diagnosed in virtually every organ system.

IgG4-RD lung involvement was first reported in 2004 by Taniguchi and colleagues in a patient with interstitial pneumonia, autoimmune pancreatitis and IgG4-positive plasma cells in the interstitium [3]. Although lung involvement has been reported in many IgG4-RD studies since then, the majority of them were not confirmed by lung biopsies. To our knowledge, the only two biopsy-proven studies of IgG4-related lung disease (IgG4-RLD) were retrospective and performed in Japan [4, 5]. However, both studies included patients diagnosed via transbronchial lung biopsy. In our opinion, the lung specimen from transbronchial lung biopsy is too small to make a pathological diagnosis of IgG4-RLD. Therefore, we made this study including 17 cases of biopsy-proven IgG4-RLD, with specimens obtained via thoracotomy, video-assisted thoracoscopic surgery (VATS) or percutaneous transthoracic core-needle lung biopsy, to better understand this rare disease.

## Methods

### Patients

Patients suspected of IgG4-RLD at Peking Union Medical College Hospital from January 2011 to July 2015 were investigated in October 2015. Only patients who fulfilled the following inclusion criteria were included in this study: (i) pulmonary parenchymal abnormality via chest computed tomography (CT) scanning; (ii) histopathological examination of a lung specimen obtained by thoracotomy, VATS or a percutaneous transthoracic CT-guided core-needle lung biopsy that demonstrated the features of IgG4-RD, including (a) marked lymphocyte and plasmacyte infiltration and fibrosis; and (b) a ratio of IgG4+/IgG+ cells > 40 % and/or > 10 IgG4+ plasma cells/HPF [6]; and (iii) histopathological examination that precluded other diseases, such as Castleman’s disease, lymphoma, or lung cancer.

Patients were defined as definite, probable or possible IgG4-RLD according to the 2011 comprehensive diagnostic criteria for IgG4-RD [6]. In order to define every patient, patients without serum IgG4 results were considered to have a normal serum IgG4 concentration. Data were collected by medical records, telephone and letters. The following data were retrieved for analysis: age, sex, clinical symptoms, laboratory findings, pulmonary function test results, chest CT tests, ^18^F-fluorodeoxyglucose positron emission tomography (^18^F-FDG PET) examinations, treatments and prognoses.

The study was approved by the Ethical Committee of Peking Union Medical College Hospital (Reference number: S-K038). Informed consent for participation and publication was obtained from all patients at the time of diagnosis or follow-up. If the patient was dead or uncontactable, the informed consent was obtained from their families.

### Statistical analysis

Data were analyzed using the Statistical Package for the Social Sciences (SPSS, version 19.0 software) (SPSS Inc., Chicago, Illinois, USA). Mean values were expressed as values + standard deviations.

## Results

### General characteristics and extrapulmonary involvement

**Fig. 1:**
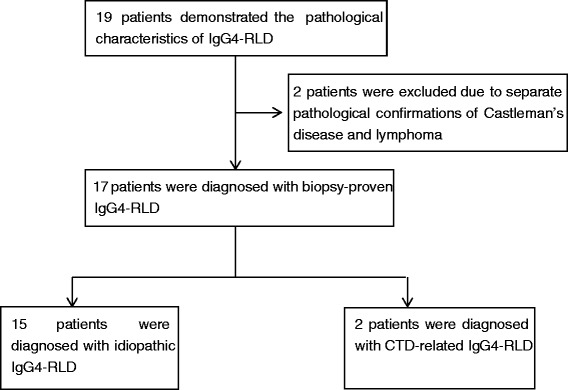
Flow diagram of the diagnostic process for IgG4-related lung disease

A total of 19 Chinese patients fulfilled the inclusion criteria (i) and (ii) and IgG4-RLD was suspected. However, 2 patients were excluded due to other malignant diseases that were pathologically discovered at the time of diagnosis. Therefore, 17 IgG4-RLD patients were included in this study. The inclusion procedure is illustrated in Fig. 1. According to the 2011 comprehensive diagnostic criteria, 7 (41.2 %) and 10 (58.8 %) patients were diagnosed with definite and probable IgG4-RLD respectively.

Among the 17 included patients, 4 patients were initially diagnosed with nonspecific interstitial pneumonia (NSIP) in the 2000s. However, a pathological review performed in 2012 confirmed a diagnosis of IgG4-RLD [7]. Two patients were diagnosed with primary Sjogren syndrome and IgG4-RLD simultaneously. IgG4-RLD was considered to be connective tissue disease (CTD) - related in both patients.

There were nearly twice as many female patients. The patient age ranged from 18 to 71 years (mean age: 44.8 years). Four patients had a history of smoking (range: 16 to 50 pack-years). Comorbidity was noted in 11 patients. Except for primary Sjogren syndrome, which was diagnosed at the same time as IgG4-RLD, the other comorbidities were all previously diagnosed (Table 1).

**Table 1:** General characteristics of 17 patients with IgG4-related lung disease

	Number (%) or mean (range)
Male : Female	1 : 1.83
Age (years)	44.8 ± 15.0 (18–71)
History of smoking	4/17 (23.5 %)
Comorbidity	
Allergy	3/17
Primary Sjogren syndrome	2/17
Healed pulmonary tuberculosis	2/17
Chronic gastritis	1/17
Diabetes mellitus	1/17
Obstructive pulmonary disease	1/17
Asthma	1/17
Vitiligo	1/17
Psoriasis	1/17
Hypertension	1/17
Clinical symptoms	
Cough	11/17 (64.7 %)
Fever	7/17 (41.2 %)
Dyspnea	5/17 (29.4 %)
Chest pain	4/17 (23.5 %)
Hemoptysis	2/17 (11.8 %)
Asymptomatic	2/17 (11.8 %)
Laboratory findings	
White blood cell count > 10 × 10^9^/L	7/17 (41.2 %)
ESR > 20 mm/h	11/17 (64.7 %)
CRP > 5 mg/L	8/17 (47.1 %)
IgG > 17 g/L	11/15 (73.3 %)
IgG4 > 1350 mg/L	7/13 (53.8 %)
IgG4/IgG > 8 %	5/13 (38.5 %)
tIgE > 100 IU/mL	5/8 (62.5 %)
ANA ≥ 1:80	3/16 (18.8 %)
Chest CT findings	
Nodules and masses with spiculated borders	8/17 (47.1 %)
Alveolar consolidations with air bronchograms	5/17 (29.4 %)
Ground glass opacities ± reticular opacities	6/17 (35.3 %)
Hilar or mediastinal lymphadenopathy	5/17 (29.4 %)
Thickening of the bronchovasular bundles	2/17 (11.8 %)
Thickening of the interlobular septa	2/17 (11.8 %)
Pulmonary function test	
FEV1/FVC < 70 %	1/9 (11.1 %)
TLC% < 80 %	3/7 (42.9 %)
DLCO% < 80 %	8/8 (100 %)

*ESR* erythrocyte sedimentation rate, *CRP* C-reactive protein, *ANA* antinuclear antibody, *FEV1* forced expiratory volume in 1 s, *FVC* forced vital capacity, *DLCO* diffusing capacity of the lung for carbon monoxide

Out of 17 biopsy-proven IgG4-RLD patients, extrapulmonary involvement was proven in only one patient with uveitis and biopsy-proven IgG4-related mastoiditis, with the help of CT and PET (lymph node less than 1 cm in diameter was considered nonspecific).

### Clinical symptoms and laboratory findings of IgG4-RLD

Most patients had mild or severe clinical symptoms (Table 1), but two patients were asymptomatic and pulmonary abnormalities were discovered accidently. The time from onset of the initial symptoms to diagnosis ranged from 1 month to 7 years, and the median interval time was 8 months.

Main laboratory findings are listed in Table 1. Serum IgG4 concentration was examined in all patients except for the 4 patients initially diagnosed with NSIP. Serum IgG4 level ranged from 77 to 7490 mg/L with a mean level of 1955 ± 1968 mg/L. Further examination revealed that the WBC, ESR and CRP levels were higher in patients with fever compared to patients without fever [WBC (×10^9^/L), 11.5 vs 8.06; ESR (mm/h), 72.7 vs 29.7; CRP (mg/dL), 85.7 vs 13.4], and patients with high serum IgG4 concentrations were more prone to have fever, compared with those with normal serum IgG4 concentrations (5/7 vs 1/6).

### Chest CT, PET and pulmonary function test results for patients with IgG4-RLD

**Fig. 2:**
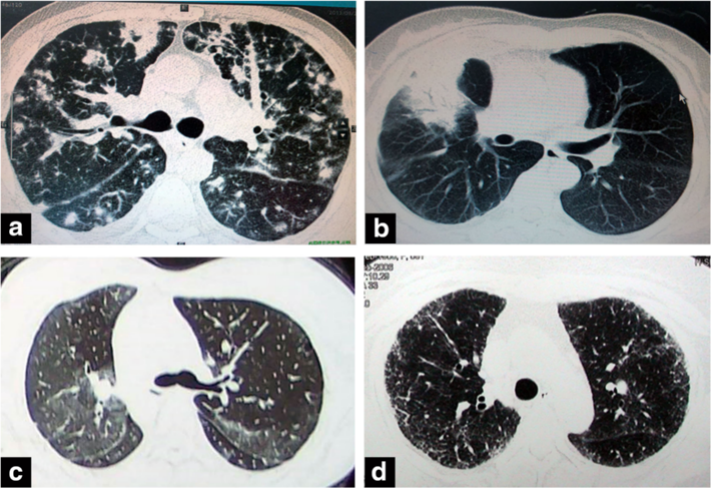
Different manifestations of IgG4-related lung disease via chest computed tomography (CT) scans. **a** Multiple nodules and masses with variable sizes are shown in both lungs via CT scans. Peribronchovascular thickening and thickened interlobular/interlobar septa were also noted. **b** Alveolar consolidations with air bronchograms in the right upper lobe. **c** Multiple ground glass opacities without reticular opacity. **d** Ground glass opacities with reticular opacities and early honeycombing

Chest CT findings and pulmonary function test results are listed in Table 1. Multifocal or diffuse lesions were observed in 13 (76.5 %) patients, and 4 (23.5 %) patients presented with local lesions. Nodules and masses with spiculated borders (Fig. 2a), alveolar consolidations with air bronchograms (Fig. 2b), ground glass opacities with or without reticular opacities (Fig. 2c, d), and hilar or mediastinal lymphadenopathy (lymph node longer than 1 cm in diameter) were the most common CT findings.

Eight patients received ^18^F-FDG PET examinations. The maximum standard uptake values (SUVs) ranged from 4.0 to 11.8 (7.4 ± 3.0). Benign inflammation was diagnosed in 7/8 (87.5 %) patients, and malignancy was suspected in only 1/8 (12.5 %) patient. During evaluation, PET showed a high SUV in sublingual gland in one patient, however, biopsy excluded IgG4-RD involvement.

### Histological findings for IgG4-RLD

A wedge resection or lobectomy was performed in 8 patients using VATS and 2 patients using posterolateral thoracotomies. Seven other patients were diagnosed using a CT-guided core-needle lung biopsy. Three patients first received a transthoracic core-needle lung biopsy and eventually underwent VATS or posterolateral thoracotomy due to nonspecific results.

**Fig. 3:**
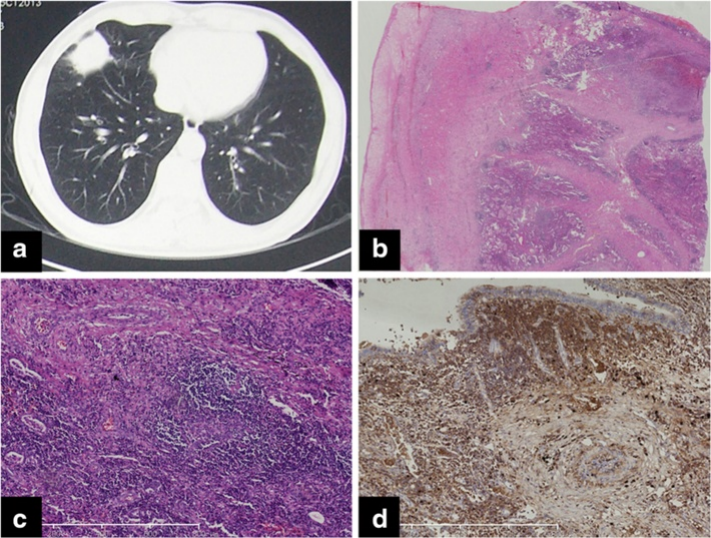
**a** Chest computed tomography scan in a 55-year-old man shows a spiculated mass in the right middle lobe. **b** Histopathological specimen obtained from this patient shows distorted alveolar structures and thickened pleura. **c** Diffuse lymphoplasmocytic infiltrates and fibrosis are observed at high magnification (H&E; ×100). **d** An increased IgG4+ cell count is identified (IgG4 immunostaining; ×100)

Histopathologically, all specimens showed diffuse lymphoplasmacytic infiltration and irregular fibrosis within the peribronchial and perivascular connective tissues (Fig. 3c). The alveolar structure was severely distorted and obliterative phlebitis was a common presentation in nodule or mass lesions (Fig. 3a, b). The IgG4+ cell count was increased in all patients (Fig. 3d), and the ratio of IgG4+/IgG+ cells was also increased. As part of the inclusion criteria, malignancy was precluded by morphological and immunohistochemical examinations.

### Treatment and prognosis

**Fig. 4:**
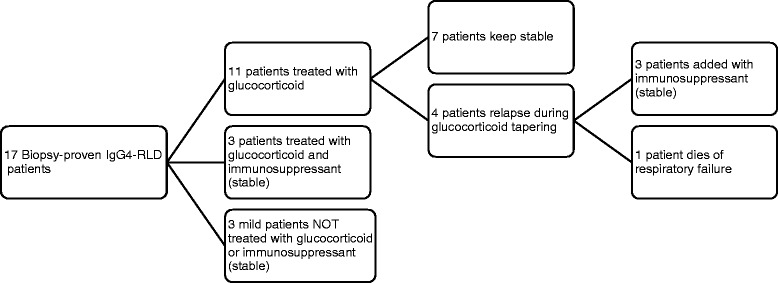
Treatment and prognosis of 17 patients with IgG4-related lung disease

Prednisone (20–50 mg per day) or an equivalent dose of another glucocorticoid was administered to 14 patients. Immunosuppressive agents were administered in 6 of theses patients, including cyclophosphamide (4 patients), azathioprine (1 patient) and mycophenolate mofetil (1 patient). During the follow-up (1 month to 8 years, median time: 18 months), clinical remission was achieved in all patients. However, relapse was observed in 4 patients during glucocorticoid tapering (Fig. 4). Persistent pulmonary abnormalities (shrunken, but similar to the initial manifestation) were observed in the 11 patients who were followed for more than 6 months.

Three patients with mild and stable lung lesions did not receive any corticosteroid or immunosuppressant, and all of them were stable (confirmed by chest CT) during the mean follow-up time of 3 years.

Death was observed in one patient, who died of respiratory failure caused by relapse of IgG4-related interstitial lung disease after glucocorticoid tapering.

## Discussion

As a newly recognized disease, IgG4-RLD is still not well understood. In this retrospective study, we analyzed 17 cases of biopsy-proven IgG4-RLD in Chinese to identify the characteristics of this rare disease.

It is still unclear how often lung involvement occurs in patients with IgG4-RD. A retrospective study of 90 patients with autoimmune pancreatitis revealed that 54 % of patients had lung lesions [8], but another study of 116 patients with biopsy-proven IgG4-RD revealed that biopsy-proven IgG4-RLD accounted for only approximately 10 % of the cases [9]. The difference might be explained by following reasons: (i) In IgG4-RD patients with multiple organs involvement, lung specimen is less probably obtained because it is more invasive; (ii) Infection, aspiration pneumonia, pulmonary congestion, etc., also contribute to lung lesions in IgG4-RD patients, which might be misdiagnosed as lung involvement. Therefore, IgG4-RLD should be cautiously diagnosed without a pathological confirmation.

Compared with typical IgG4-RD involving two or more organs, this study showed uncommon extrapulmonary involvement in biopsy-proven IgG4-RLD, even with the help of PET. Although selection bias exists because patients with only lung involvement are more prone to receive a lung bi,opsy, this association indicates that lung involvement is probably over-estimated in IgG4-RD patients. Thus, pathological confirmation seems crucial for the diagnosis of IgG4-RLD.

The etiology of IgG4-RLD is still unknown. Autoimmunity is currently considered as the main pathogenic mechanism [10]. Two patients were simultaneously diagnosed with primary Sjogren syndrome in this study, proving the relationship between IgG4-RLD and CTDs. An antibody against plasminogenbinding protein of *Helicobacter pylori* has been identified in more than 90 % of patients with autoimmune pancreatitis [11], proposing that infectious agents may play a role in the pathogenesis of IgG4-RD. But in our study, the association between IgG4-RLD and infection was not identified.

The clinical symptoms of IgG4-RLD depend on the lesion locations and are usually nonspecific [12]. In our study, cough, dyspnea, chest pain and hemoptysis were the primary respiratory symptoms. Fever was observed in nearly half of the patients in this study indicating that constitutional symptoms are not as rare as previously thought [13]. Although asymptomatic patients were also noted in our study, this phenomenon was not as common as reported in a previous Japanese study [4].

One study of IgG4-RD patients demonstrated that serum IgG4 concentrations > 1350 mg/L and serum IgG4/IgG ratios > 8 % had a sensitivity of 97.0 and 95.5 %, respectively [14]. However, in our study, the above criteria had a sensitivity of only 53.8 and 38.5 %, respectively, in IgG4-RLD patients. Serum IgG4 concentrations tend to be higher in patients with multiple organ involvement [15]. Most patients in this study had only lung involvement, which may explain the discrepancy. Therefore, it is not appropriate to exclude a diagnosis of IgG4-RLD due to a normal serum IgG4 concentration. Inflammatory marker concentrations are higher in IgG4-RLD patients with fever, suggesting that constitutional symptoms are associated with disease severity. This phenomenon has been previously reported by our team [16]. More aggressive treatment is usually necessary in these patients.

Inoue and colleagues summarized four patterns of CT findings: solid nodular, round-shaped ground glass opacity, alveolar interstitial, and bronchoalveolar [17]. However, in our study, lobar or segmental consolidation was commonly seen. Ground glass opacity was patchy or diffuse. A bronchoalveolar manifestation was usually mixed with other more prominent patterns. Therefore, we prefer the following categories based on the main manifestation: (i) nodule or mass with spiculated margins; (ii) lobar or segmental consolidation with air bronchograms; (iii) ground glass opacity; (iv) alveolar interstitial; and (v) bronchovascular. However, a mixed pattern is commonly seen in IgG4-RLD patients. Mediastinal and/or hilar lymphadenopathy have been described in 40 to 90 % of patients with IgG4-RD in literature [13], however, a lower rate of mediastinal or hilar lymph node involvement is found in this study, which may be explained by the reason that a restricted definition of lymphadenopathy is applied in this study, and might be associated with only one organ involvement in most cases.

The use of ^18^F-FDG PET uncovers more organ involvement than conventional evaluations in IgG4-RD patients [18]. In IgG4-RD patients with involvement of two or more organs detected by PET, lung involvement occurs in approximately 25 % of cases [18, 19]. However, abnormal uptake is not always associated with IgG4-RD involvement [19], as demonstrated in our study. Therefore, the explanation of a high SUV should be cautious in IgG4-RD patients.

Although lung tissue obtained via a less-invasive, CT-guided, transthoracic core-needle biopsy is convenient, it failed to yield a definitive diagnosis in about one third of patients in this study. Thoracotomy or VATS can obtain more lung tissue, and are usually required to make a pathological diagnosis of IgG4-RLD. Transbronchial cryobiopsy is a new technique that allow to obtain larger and more qualified specimen than forceps biopsy. The use of cryobiopsy in diagnosing interstitial lung disease has been proved to be proper and promising [20, 21]. However, the value of cryobiopsy in IgG4-RLD is still to be investigated.

To treat IgG4-RLD, corticosteroids are still the cornerstone therapy. The exact regimen for corticosteroids is still not detailed, but a regimen similar to extrapulmonary IgG4-RD therapy, such as during autoimmune pancreatitis, is recommended [1]. In this study, all patients treated with steroids achieved remission, which confirmed the effectiveness of steroids for IgG4-RLD patients. Data regarding the use of immunosuppressants for IgG4-RLD is still sparse. Limited data and our study demonstrated positive results. Self-limitation has been occasionally observed in some IgG4-RLD patients [22], and was also observed in our study. We recommend medical treatment in most patients. Only some specific patients can watch and wait, and long-term follow-ups are necessary.

The value of this study is limited by the small number of included patients. Our study show that although IgG4-RLD and typical IgG4-RD share common features, IgG4-RLD has its own distinct characteristics.

## Conclusions

Biopsy-proven IgG4-RLD is different in many ways from typical IgG4-RD. In biopsy-proven IgG4-RLD, a normal serum IgG4 concentration is commonly seen, while extrapulmonary involvement is infrequent. Alveolar consolidation with air bronchograms is an important imaging finding of IgG4-RLD, which has not been emphasized before.

## Availability of data and materials

Data are available from the corresponding author upon request.

## Abbreviations

^18^F-FDG PET: ^18^F-fluorodeoxyglucose positron emission tomography
CRP: C-reactive protein
CT: computed tomography
CTD: connective tissue disease
ESR: erythrocyte sedimentation rate
IgG4: immunoglobulin G4
IgG4-RD: IgG4-related disease
IgG4-RLD: IgG4-related lung disease
SUV: standardized uptake value
VATS: video-assisted thoracoscopic surgery
WBC: white blood cell count
